# Neuromodulation Applied to Diseases: The Case of HRV Biofeedback

**DOI:** 10.3390/jcm11195927

**Published:** 2022-10-08

**Authors:** Asaf Gitler, Leen Vanacker, Marijke De Couck, Inge De Leeuw, Yoram Gidron

**Affiliations:** 1 Faculty of Social Welfare and Health Sciences, Department of Nursing, University of Haifa, Haifa 3498838, Israel; 2 Faculty of Medicine and Pharmacy, Department of Public Health, Mental Health and Wellbeing Research Group, Free University of Brussels (VUB), 1140 Evere, Belgium

**Keywords:** vagus nerve, heart-rate-variability (HRV), vagally mediated HRV (vmHRV), biofeedback, hypertension, coronary heart disease, cancer, pain, clinical outcomes

## Abstract

The vagus or “wandering” nerve is the main branch of the parasympathetic nervous system (PNS), innervating most internal organs crucial for health. Activity of the vagus nerve can be non-invasively indexed by heart-rate variability parameters (HRV). Specific HRV parameters predict less all-cause mortality, lower risk of and better prognosis after myocardial infarctions, and better survival in cancer. A non-invasive manner for self-activating the vagus is achieved by performing a slow-paced breathing technique while receiving visual feedback of one’s HRV, called HRV-biofeedback (HRV-B). This article narratively reviews the biological mechanisms underlying the role of vagal activity and vagally mediated HRV in hypertension, diabetes, coronary heart disease (CHD), cancer, pain, and dementia. After searching the literature for HRV-B intervention studies in each condition, we report the effects of HRV-B on clinical outcomes in these health conditions, while evaluating the methodological quality of these studies. Generally, the levels of evidence for the benefits of HRV-B is high in CHD, pain, and hypertension, moderate in cancer, and poor in diabetes and dementia. Limitations and future research directions are discussed.

## 1. Introduction

One of the goals of medicine is to identify risk and protective factors of diseases, to prevent negative health consequences, and promote health. It is also crucial to reveal the underlying biological mechanisms leading to health. Therefore, it seems important to reveal the variables and interventions which promote well-being rather than diseases.

The leading causes of death worldwide include coronary heart disease (CHD), cancer, stroke, and chronic obstructive pulmonary disease (COPD) [1]. When considering the amount of disability due to health conditions, chronic pain, anxiety, and depression are added to this list of GBD [2]. Given their prevalence and costs for the individual and society, these call for identifying common risk factors and relatively simple and inexpensive evidence-based interventions to reduce GBD and their associated disabilities, to promote longevity with well-being.

Ongoing communication between vital organs through the autonomic nervous system (ANS) is a necessary feature of a healthy organism [3], and it has been shown that synchronization between neurophysiological and peripheral disease markers has prognostic value [4]. The ANS is composed of the sympathetic nervous system (SNS) and parasympathetic nervous system (PNS) and plays a crucial role in preserving homeostasis in response to both internal and external stimuli [5].

The vagus or “wandering” nerve is the main branch of the PNS, innervating most internal organs which have crucial roles in health and illness (e.g., heart, lungs, liver, colon). The vagus nerve is the 10th cranial nerve that extends from the brain stem and then near the left and right carotid arteries to innervate most visceral organs. Approximately 20% of its nerve fibers are efferent, while the majority (80%) are afferent, sending various signals from visceral organs to the brain [6]. Anatomically, descending vagal fibers arise from the nucleus ambiguous and the dorsal vagal nucleus of the brainstem and synapse at the cardiac plexuses and cardiac ganglia, which innervate the sinoatrial node, the atrioventricular node, the coronary arteries, and the ventricular myocytes [7].

The vagus has a major neuro-immunological role. The nervous and immune systems constantly influence each other bidirectionally. The nervous system innervates lymphoid glands and immune cells express receptors for neurotransmitters [8,9]. The immune system communicates with the brain via direct penetration of the brain in regions lacking the blood–brain barrier (BBB), via monocyte “cross-talk” on both sides of the BBB, and via the vagal nerve [10,11].

The vagal nerve transmits information about peripheral inflammation via receptors for interleukin-1 on its paraganglia, and then it regulates peripheral inflammation by two routes. First, it activates the hypothalamic–pituitary–adrenal (HPA) axis, leading to the secretion of cortisol which decreases inflammation. Second, the descending vagus reaches the celiac ganglion where it “converts” to a sympathetic branch that innervates the spleen. There, a sub-class of splenic T-cells produce in return the vagal neurotransmitter acetylcholine, which binds to its alpha-7 nicotinic acetylcholine receptor on macrophages, which inhibits inflammatory cytokine synthesis [12]. These relatively unknown neuro-immuno-modulatory roles of the vagus have crucial implications for disease control, as we shall see below.

One more important parasympathetic role of the vagus is vasodilation which counteracts the sympathetic response. This is achieved specifically by vagal nerve-induced increases in vasoactive intestinal peptide, which then increases coronary blood flow [13]. This anti-hypoxic role is crucial for reducing the risk of CHD, stroke, and also even cancer since many tumors flourish in hypoxic conditions and hypoxia is prognostic in cancer [14].

The heart rate rises during inhalation and falls during exhalation [15], due to increased vagus nerve activity during exhalation. This influence of the PNS on cardiac activity that occurs via the vagus nerve permits the physiological mechanism of respiratory sinus arrhythmia (RSA). RSA reflects oscillations in heart rate (HR) in correspondence to breathing. It should be noted that the respiratory rate is an independent predictor of mortality in CHD [16]. The vagus, when activated, reduces the respiratory rate.

## 2. Heart Rate Variability—The Heart’s Eye to the Vagus

The activity of the vagal nerve is non-invasively indexed by different HRV parameters which reflects the fluctuations in the intervals between normal heart-beats [17]. HRV mostly reflects the efferent vagal influence on the heart and can be measured from an ECG or from intervals in pulses on the ear lobe, chest, or index finger using photoplethysmography. HRV is strongly correlated with actual vagal nerve activity (r = 0.88) [18]. Specifically, HRV indexes the activity of the vagus nerve regulating cardiac function termed cardiac vagal activity or cardiac vagal tone [19]. The HRV parameters suggested that reflect cardiac vagal activity are often referred to as vagally mediated HRV (vmHRV). Higher levels of vmHRV indicate greater vagal tone, which also reflects the body’s ability to respond to increased metabolic demands and environmental challenges. Key parameters that reflect this are RMSSD and HF (measured and presented in ms and ms^2^, respectively) as we will elaborate on below.

HRV is modulated by multiple regulatory mechanisms which operate at different time scales. A 24-h recording of HRV reflects the interactions between circadian rhythms, core body temperature, metabolism, and vagal efferent signals to the heart. In contrast, short-term measurements of HRV (i.e., 5 min) reflect autonomic, cardiovascular, and respiratory effects, and are also a valid marker of parasympathetic cardiac vagal control as mentioned [17,20].

HRV can be detected using two major different linear domains: frequency and time domains.

In the frequency domain, HR oscillations are divided into four frequency bands: ultralow frequencies (ULF ≤ 0.003 Hz)); very-low frequencies (VLF: 0.003–0.04 Hz); low frequencies (LF: 0.04–0.15 Hz); and high frequencies (HF: 0.15–0.40 Hz). The ULF band indexes fluctuations in the inter-beat interval (IBI) with a period from 5 min to 24 h and is usually measured using 24 h recordings and reflects slow biological processes such as circadian rhythms and metabolism. The VLF band reflects physical activity, temperature regulation, and PNS and SNS control and is comprised of rhythms with periods between 25 and 300 s. The LF band reflects baroreflex control and is mostly controlled by the PNS, and to a lesser extent by the SNS. LF is comprised of rhythms with periods between 7 and 25 s and is affected by breathing from 3 to 9 bpm. The HF band reflects changes in heart rate due to respiration and is fully controlled by the PNS. Since the influence of the PNS on the HRV occurs via the vagus nerve, this permits the physiological mechanism of RSA. In normal conditions, the RSA reflects oscillations in HR in correspondence with breathing and very strongly correlates with the HF parameter. The HF domain is influenced by breathing from 9 to 24 bpm [17]. The ratio of LF to HF is called the LF/HF ratio, and its meaning is controversial, especially regarding the term ′sympatho-vagal balance′. Claims regarding the LF/HF ratio reflecting the ′sympatho-vagal balance′ are generally rejected by researchers [21,22,23]. In contrast to initial thinking, it has been shown that LF-HRV is not a reliable indicator of sympathetic nervous activity [21,24,25]. Consequently, the sympatho-vagal balance as measured by the LF/HF ratio is an inaccurate conceptualization [22,23]. Different processes appear to generate 24 h and 5 min values, and these values correlate poorly. Furthermore, the SNS contribution to LF power varies profoundly with testing conditions and interpretation of baseline LF/HF ratios depends highly on specific measurement conditions [26]. Empirical evidence also suggests that the LF and LF/HF ratio primarily reflect vagal PNS control [27]. Total power measure is the sum of the energy in the ULF, VLF, LF, and HF bands, and it can also be calculated for 24 h. The VLF, LF, and HF bands are calculated over short-term recordings [17,27].

In the time domain, the amount of variability in the IBI between successive heartbeats is quantified by several indexes (SDNN, SDANN, pNN50, HR Max-HR Min, RMSSD, etc.). Although HRV guidelines recommend that measurements be based on a minimum of 5-min recordings, the ultra-short measurement of 10-s ECGs are more commonly made during routine medical care and are thus more patient friendly. Furthermore, a 10-s signal is suitable for obtaining the two most commonly used time domain HRV indexes: the standard deviation of the normal-to-normal RR intervals (SDNN) and the root mean square of successive RR interval differences (RMSSD) [17]. Importantly, there are strong correlations between 10-s and 5-min pulse-wave recordings for RMSSD, and to a lesser extent for SDNN [28]. HRV markers obtained from 10-s ECGs have been associated with heart failure [29], and even prediction of all-cause mortality [30], and cancer prognosis [31]. People with higher RMSSD were found to recover faster from stress on hormonal (cortisol), inflammatory (TNF), and cardiac (DBP) parameters [32], and neurobiological evidence suggests that HRV (both time and frequency indices) is impacted by stress and supports its use for the objective assessment of psychological health and stress [33]. Moreover, the frequency domain measure HF-HRV can be used as a biomarker of psychopathology [34]. The above demonstrates the vast role of the vagus in psychobiological resilience. This could have significant health implications since many diseases are also predicted by mental stress [35,36].

It is also important to note that vagal activity influences and is controlled by higher brain regions. According to the neurovisceral integration model [37], vmHRV is associated positively with a large range of positive outcomes including executive functions, emotion, and health, displaying better self-regulation of the organism [37,38]. Several brain regions, involved in perceptions of threat and safety and in emotional regulation, are also associated with different HRV measures. Meta-analyses showed that with vmHRV, both frequency and time domain measures were associated with activity in the amygdala, various sections of the cingulate, and the prefrontal cortex, showing how a prefrontal-guided “core integration” system is integrated with the brainstem nuclei which regulate the heart [33,38].

Some researchers have investigated the relationship between time and frequency variables. For example, SDNN correlated with LF power, and RMSSD correlated with HF power during sleep in men [39]. RMSSD is highly correlated with high frequency [40]. The time domain index RMSSD obtained in short-term RR segments was not affected by changes in respiration [41], however, respiration was associated with HF [42].

## 3. The Role of HRV in Health and Diseases

This article will show in depth the prognostic role of frequency and time domain HRV measures in multiple health outcomes. Indeed, high HRV levels measured very-low-frequency power (*p* < 0.0001), low-frequency power (*p* < 0.0001), high-frequency power (*p* = 0.0014), total power (*p* < 0.0001), and the standard deviation of total normal RR intervals (*p* = 0.0019) were significantly associated and predicted less all-cause mortality [43]. An SDNN less than 70 msec compared to patients with an SDNN > 70 msec showed a far better prognosis after myocardial infarctions (MI). Meta-analysis demonstrates that, after a MI, patients with an SDNN below 70 msec on 24 h ECG recording have almost four times greater chance of dying in the next 3 years [44]. In cancer, the mean SDNN of cancer patients’ samples was significantly lower (SDNN = 22 ms) compared to a healthy sample (SDNN = 50 ms) [45]. The SDNN value can predict survival of patients with cancer, and higher vagal nerve activity predicts longer survival [46]. Reduced time and frequency domains of HRV were also observed in several chronic diseases and in people with poor health outcomes, e.g., hypertension [47,48], systematic inflammation [49,50], and diabetes [51]. Publications show an abundance of evidence for the significant prognostic value of HRV parameters, but the broad spectrum of correlated diseases and influencing factors suggest that it might have low specificity in the distinction of a specific disease. These associations can be explained by several biological mediators [52]. One of the most important variables in that regard is inflammation which plays a major role in many diseases including heart disease and cancer [53]. Many studies have shown a negative relationship between HRV parameters and inflammation across age groups [50,54,55,56]. Moreover, a prospective study [57] found that baseline HF-HRV was associated with lower levels of CRP and the predicted level of the inflammatory marker 4 years later. Thus, vmHRV, the vagus nerve index, is a marker of multiple psychobiological and neurophysiological processes and could be used to predict the onset and prognosis of major global disease burdens. Moreover, there are published norms in the literature obtained after years of ongoing research regarding short-term and ultra-short measures of HRV that covers a full-age spectrum and a variety of conditions [58,59,60,61].

## 4. Activating the Vagal Nerve by Heart Rate Variability Biofeedback

Several non-pharmacological methods exist for activating the vagus nerve. First, electric invasive and also transcutaneous and non-invasive vagus nerve stimulation (nVNS) devices have been developed. The nVNS devices were found to reduce chronic headaches [62,63], to reduce inflammation [64], and depression, as well as the prevalence of atrial fibrillation attacks [65].

The second, a non-invasive manner of self-activating the vagus, is achieved by performing slow-paced breathing while receiving feedback on one’s HRV, called HRV-biofeedback (HRV-B). This non-invasive way to increase vagal nerve activity aims to reduce breathing rate to approximately six breath cycles per minute [66]. Such deep and slow-paced breathing, as opposed to the normal respiratory rate for healthy adults which is between 12–20 breaths per minute, increases HRV specifically by using frequency domain activity for measurement and feedback. The method can easily be carried out, is inexpensive, has little to no side-effects, and there are published protocols [67,68].

Generally, breathing education or training emphasizes decreased respiration rate and increased muscle relaxation of the thoracic area, as well as training in abdominal breathing, also called diaphragmatic breathing (DB). Muscle tension in the abdominal area, pelvis, and lower back tends to restrict movement of the diaphragm, thus requiring the use of the accessory muscles of the chest and shoulders for breathing [69]. This emphasizes the fact that inefficient breathing uses an excess of muscles and tension that contributes to sympathetic arousal. Associated with this is the fact that thoracic breathing contributes to hyperventilation (over breathing resulting in lowered end-tidal CO_2_). The occurrence of hyperventilation is known to be conjoined with psychological stress [70], which contributes to an array of somatic and behavioral symptoms [71]. Slow-paced breathing has health benefits for prevention and adjunct treatment purposes with few adverse effects expected and can be advised as a preventive and adjunct treatment for many conditions [72]. Progressive muscle relaxation (PMR) includes systematically tensing and relaxing muscle segments in the body, and this targets sympathetic arousal. PMR influences the immune system as seen in studies on decreasing cortisol levels and increased NK-cells following PMR [73,74,75,76]. In contrast, HRV-B training plays an even more direct role in autonomic modulation, as it specifically targets reflexes that modulate autonomic activity via the parasympathetically controlled vagal nerve.

Biofeedback in general includes the measurement and the presentation of audio-visual feedback of biological parameters such as brain activity, muscle activity, heart rate, and measures of skin sweat glands. Biofeedback empowers users to attain control over physiological processes in conjunction with changes to their psychological state.

HRV-B specifically teaches people to exhibit optimal breathing patterns and frequency which greatly increase vmHRV while stimulating various autonomic and respiratory regulatory reflexes. As mentioned, HRV-B teaches people to breathe slowly at the rate of about six breaths per minute (a slightly different rate for each person) [77] which is slow, as mentioned, compared to the normal breathing rate among healthy adults. In this exercise, heart rate oscillations are in phase with respiration, such that heart rate rises and falls along with the pattern of the tidal breath [78,79,80]. When heart-rate changes are synchronized with changes in breathing, this is a state of cardiac, respiratory, and blood pressure coherence also referred to as HRV coherence. In that state, the frequency domain measures usually reflect a dominance of the LF band (mainly around 0.1 Hz) which is comprised of rhythms with periods between 7 and 25 s and is affected by breathing from 3 to 9 bpm, while HF and VLF decrease, and mean HR tends to decrease. HRV-B training is typically guided by a visual and auditory representation of one’s heartbeat and respiration rate in real time, to support and reinforce the process of increasing the amplitude of LF-HRV frequency power [81].

## 5. Resonance Frequency Breathing

It has been proposed that each individual’s cardiovascular system has a unique resonance frequency, which is caused by the delay in the baroreflex [82]. Inertia in the blood supply accounts for most of this delay. Breathing, rhythmic muscle tension, and emotional stimulation can activate the cardiovascular system’s resonance properties at this frequency. When clients breathe at this rate, which varies in adults from 4.5–6.5 breaths per minute, they systematically activate the baroreflex [79,83]. The baroreflex is the vagally controlled negative feedback system that controls blood pressure by modulating HR. When HR and respiration are synchronized, this results in efficient gas exchange and oxygen saturation [80,84,85]. With practice enhanced with biofeedback, clients can learn to breathe at their resonance frequency. This, as mentioned, aligns the three oscillators, namely baroreflex/blood pressure, HR, and respiration at that frequency and moves the peak frequency from the HF range (0.2 Hz) to the LF range (0.04–0.15 Hz). Breathing at the resonance frequency strongly increases the amplitude in the LF band mainly around the 0.1 Hz frequency which represents a rhythm of six breath cycles a minute, while each cycle of inhalation and exhalation comprised of approximately 10 s. HRV biofeedback training immediately produces large-scale increases in baroreflex gain, and after weeks and months of steady practice, this increased gain continues even when clients are not receiving feedback [67]. Increased baroreflex gain means that the cardiovascular system produces large-scale HR increases and decreases when a client inhales and exhales, respectively.

Resonance frequency breathing using HRV-B may increase the cholinergic anti-inflammatory pathway [86,87]. One pilot trial found that compared to a control group that completed HRV-B but for participants’ usual respiration rate, those who completed HRV-B in resonance frequency had reductions in CRP [88]. Though based on a small sample (*n* = 10), this was a rigorous and important experiment.

Clinically significant effects of HRV-B have been found for a wide variety of disorders. Several meta-analyses and systematic reviews examined the effects on stress and anxiety, depression, and emotional and physical health [89,90,91]. Their main conclusions were that HRV-B training is associated with a large reduction in self-reported stress and anxiety, and improves depressive symptoms and functioning. Another recent meta-analysis found significant positive effects on hypertension, cardiovascular prognosis, inflammatory states, and pain [92].

The effectiveness of HRV-B interventions is well established in the field of mental health with an emphasis on anxiety and depression, while a focus aimed at examining the effect on major physical diseases is lacking. In addition, many reviews of HRV-B in various illnesses often lack a deeper explanation of the biological mechanisms linking vagal nerve activity with the particular illness. The current review article aimed to address these two important gaps.

## 6. The Role of the Vagus Nerve and HRV-B in Diseases

Although this is a narrative review, we chose to focus in this chapter on presenting research findings using a structured search. The following section will briefly review, in relation to selected major health conditions, the relationships between different HRV parameters and the etiology or prognosis of each condition, the biological mechanisms linking vagal activity to each illness, and will review studies that tested the effects of HRV-B on patients with each condition. For the latter, we searched PUBMED using the following keywords: “Condition” AND (HRV OR heart rate variability) AND (trial OR RCT) AND Biofeedback. “Condition” refers to diabetes or hypertension or cancer or pain or CHD or cognitive impairment, dementia, or Alzheimer′s disease. This search was completed separately for each condition.

Concerning the effects of HRV-B, we also critically evaluated the methodological level of each identified trial per condition, using a quality assessment instrument for randomized trial studies [93]. An evaluation was completed independently by two critical appraisers with attention to the following standards and principles: 1. Were the inclusion criteria clear? 2. Were the exclusion criteria clear? 3. Were groups similar on important prognostic factors? 4. Was the intervention given in the same way to all participants in the experimental group? 5. Did participants of all groups all receive the usual treatment as well? Each answer received 0 = no; 1 = yes, hence the total methodological score raged from 0–5. The results are presented in Table S1.

## 7. Heart Diseases

First, reduced HRV predicts an increased risk of coronary heart disease. In a prospective study on 2501 initially heart-disease-free participants, reduced SDNN was an independent predictor of an excess of 47% risk for future cardiac events [43]. Second, as mentioned above, a meta-analysis of 21 studies found that patients after a myocardial infarction (MI) with an SDNN below 70 ms had approximately four times the risk of mortality, compared to those with SDNN > 70 ms [44]. More recently, in [94] a review of nine studies showed that following ST-elevation myocardial infarction (STEMI), low time domain HRV and high LF/HF ratio predicted all-cause mortality and major adverse cardiovascular events.

Oxidative stress, excessive sympathetic activity, and inflammation all contribute to atherogenesis since oxidative stress results in oxidized LDL cholesterol, and inflammation causes the recruitment of macrophages to an evolving cardiac plaque, which engulfs oxidized LDL and further increases coronary occlusion [95,96]. Finally, sympathetic over-activity also contributes to the risk of MI by multiple mechanisms including increased oxygen demand, coronary lesions, and vasoconstriction. In contrast, the vagus may slow the development of atherosclerosis and reduce the risk of subsequent MI since the vagus reduces oxidative stress, sympathetic activity, and inflammation [12,52,97].

We examined first the evidence for the effects of HRV-B on people with MI and CHD. First, in 54 CAD patients, those randomized to receive HRV-B showed increases in HRV and reductions in hostility [98], both independent prognostic factors in MI [44,99]. Another HRV-B RCT among CHD patients, [100], found a significant increase in the percentage of LF-HRV as well as an increased SDNN. In an RCT of *n* = 46 post-MI patients, half underwent HRV-B, and half did not. HRV-B led to short-term increases in HRV (SDNN, HF-HRV) and to increased self-efficacy, while this did not occur among controls [101]. In a study on 210 Chinese CAD patients, half underwent HRV-B, half not, and all were followed for 1 year. Only following HRV-B did the LF-HRV significantly increase, and readmissions to the hospital decreased at the 1-year follow-up [102]. Another RCT of HRV-B plus cognitive behavioral stress management training, [103], found that improved psychological adjustment was significantly associated with the HF index of vagal HR modulation only in the HRV biofeedback group. All studies achieved a methodological score of 5. Thus, concerning the outcomes of HRV, psychological well-being, and readmission rates, there is a high level of evidence for the effectiveness of HRV-B in patients after MI, or with CAD and CHD.

## 8. Hypertension

Epidemiologically, multiple studies have shown inverse relationships between HRV and blood pressure (BP), both cross-sectionally and in prospective studies. For example, a study conducted in the Atherosclerosis Risk in Communities (ARIC) study found in 7099 people initially without hypertension (HT) that reduced SDNN and RMSSD significantly predicted an increased risk of HT over 9 years [104].

The vagal nerve is crucial in the evolution of hypertension since this nerve plays a pivotal role in regulating blood pressure (BP) via the baroreflex. This reflex is based on mechanoreceptors in large vessels such as the aorta which detects intra-arterial pressure. To counteract this, the vagus then commands a reduction in HR via the brain stem, which then reduces cardiac output, resulting in the reduction in BP. In addition, BP may increase due to vasoconstriction, which is also counteracted by the vagus as it is a vasodilator.

In an important RCT, 43 people with pre-hypertension received either HRV-B, deep breathing training without biofeedback, or no treatment, and were followed for 3 months. Significant increases in HRV and reductions in SBP and DBP were only found in the HRV-B group [105]. That study also demonstrated the importance of receiving biofeedback beyond merely performing paced breathing. Biofeedback could serve both as a reinforcement and as a self-monitoring process. In another RCT with 65 hypertensive patients, HRV-BF but not an autogenic relaxation (control) led to reduced SBP [103]. In another examination of HRV-B among hypertensive patients conducted among 22 patients, 12 participants with high blood pressure and 10 with low blood pressure were included [106]. No control was included. All participants performed three individual sessions of biofeedback of the R-wave-to-pulse interval biofeedback (not precisely HRV-B but conceptually similar) over a 2-week period. Participants with high blood pressure achieved significant reductions in diastolic blood pressure levels from the beginning of the first to the end of the last training session. In contrast, participants with low blood pressure achieved significant increases in diastolic blood pressure levels. However, since no control groups were included for either sub-group, the results of this study are difficult to interpret.

The mean methodological evaluation score for these studies in HT was quite high–4. Thus, for HT, the evidence for the positive effects of HRV-B on BP control is quite strong.

## 9. Pain

The relationship between vagal nerve activity and pain is complex, yet quite convincing in multiple dimensions of evidence. First, SDNN is negatively and significantly correlated with pain [107]. In a recent study, the authors of [108] found a profound negative correlation (r = −0.81) between pre-surgical HF-HRV and postoperative pain after epileptic surgery. That relationship also remained significant after statistically controlling for multiple confounders.

Biologically, pain takes place due to vasoconstriction causing ischemia, oxidative stress found in some pain conditions [109], inflammation [110], and excessive sympathetic activity [111]. Again, the vagus inhibits all three and its activity partly negates the brain pattern termed ′the brain pain matrix′′ [112].

Does HRV-B reduce pain? Several RCTs do provide such evidence. A pilot RCT allocated 14 patients with chronic pain to HRV-B or to usual care only. After treatment, the HRV-B evidenced lower pain than controls [113]. Another RCT sampled 24 patients with ′′stress-induced′′ chronic neck pain, of whom half received HRV-B. HRV-B led to improved pain, vitality, and social functioning [114]. Another RCT examined 21 children aged 8–17 with chronic pain [115], and found significant reductions in self-reported pain intensity and higher levels of self-reported school functioning after HRV-B training. The mean methodological evaluation score of these three RCTs was 5, demonstrating strong methodological rigor. These findings also demonstrate that, beyond treating pain, HRV-B also appears to help patients′ functioning in society, seen both in children and adults.

## 10. Cancer

In cancer, HRV parameters predict better prognosis (lower tumor marker levels and lower risk of death), independent of confounders, according to two systematic reviews and one meta-analysis [46,116,117]. Furthermore, the HRV–survival relationship was found to be statistically mediated by reduced inflammation, specifically in pancreatic cancer [116].

Various systemic and local factors in the tumor microenvironment promote tumorigenesis including oxidative stress [118], inflammation [53], sympathetic hyperactivity [119], and hypoxia [120]. Concerning sympathetic activity, tumors appear to metastasize to regions rich in norepinephrine [119]. In contrast, anti-tumor immunity (e.g., CD8+ and NK cells) predict a good prognosis in cancer [121].

However, the vagal nerve inhibits oxidative stress [122], “reflexively” reduces inflammation [12], reduces sympathetic activity (being the major branch of the parasympathetic nervous system [123], and its activity should inhibit hypoxia because the vagal nerve induces vasodilatation [124]. Furthermore, the vagus increases NK and CD8+ cells [125], which also have anti-tumor effects. Since the vagus alters all these factors, its activity is thought to slow tumorigenesis [126,127].

A few studies have tested the effects of HRV-B in cancer, yet mostly on the “symptom cluster”–pain, distress, fatigue, and depression. One feasibility RCT with 31 patients randomized cancer patients to HRV-B for up to 6 weeks, or to a wait-list control. HRV-B increased HRV-coherence and reduced insomnia, fatigue, pain, and distress [128]. In another RCT of 34 patients with different cancers, half underwent 4–6 weeks of HRV-B. HRV-B led to increases in HRV indices but in particular to improved sleep [129]. An open-label, comparative study [130] examined 50 patients with incurable cancer and sleep disturbance; HRV-B sessions followed by daily home-based practice for approximately 2 weeks again led to an improvement of sleep efficiency and to increased LF-HRV. Another study on 17 patients with hematological malignancies in a pre-post study design with no control group [131], examined HRV-B for over 12 weeks, with 20 min of daily home practice together with supervised sessions of physical exercise. Significant improvement was found in physical capacity, muscle strength, and flexibility, together with increased LF-HRV. However, since no control group was included in this two-component intervention, we cannot differentiate the effects of the HRV-B from those of the physical activity. In a small sample, five patients with primary brain tumors were studied [132], a pre-post intervention study without a control group, significant reductions in depression and anxiety measures were found.

**Figure 1:**
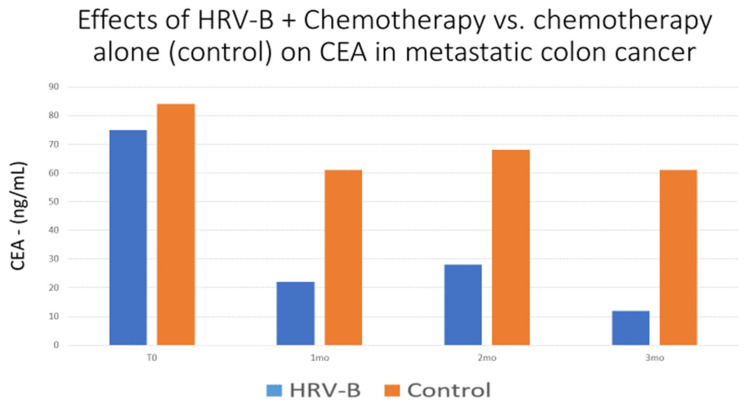
Effects of heart-rate variability biofeedback + usual medical care (HRV-B) versus usual medical care alone (controls) on the tumor marker CEA across time (in months) in a pilot-matched controlled study.

In a pilot-matched controlled study executed on a very small sample [133], three patients with metastatic colon cancer underwent HRV-B, daily home-based for about 20 min/day, for 3 months, in addition to usual care. Controls only received usual treatment. These were matched on tumor type (colon) and stage (4), type of chemotherapy, and baseline CEA levels to three controls. As shown in Figure 1, patients in the HRV-B evidenced sharp reductions in CEA levels, while controls did not. However, that study included only six patients and it was not an RCT. Nevertheless, it was a well-matched controlled study and its results strongly call for replication on a large scale. The mean score on the methodological evaluation was 3.8. Thus, here the evidence for HRV-B and subjective outcomes is high, while for physical outcomes as well as oncological outcomes, the evidence is low, in addition to the medium level of methodological evidence. These cast doubt on the inferences concerning the effects of HRV-B on oncological outcomes including recurrence, survival, and quality of life. This gap is in sharp contrast to the high-level evidence linking HRV to prognosis in cancer.

## 11. Diabetes

In a review of 25 case-control studies, HRV was reliably lower in type-2 diabetes than in healthy controls [51]. The relationship between HRV and HbA1C, the marker of average glucose levels over 2–3 months, is more complex. That review found that HbA1C was inversely related to the length of R-R ECG intervals, which is the basis of HRV [51]. In addition, studies have shown a moderately strong inverse correlation between HRV and HbA1C [134]. These associations can be explained by the cholinergic anti-inflammatory effects of the vagus [11,12], and the key role inflammation plays in insulin resistance, the hallmark of diabetes [135].

Effects of HRV-B on type-2 diabetes have scarcely been tested. In one non-controlled pilot study, 17 patients with diabetes and neuropathy received 8 weeks of HRV-B training. Patient compliance was 50% and, in some patients, some HRV indices improved [136]. However, this was not an RCT and no control group was included, nor was there any measure of insulin control. Another non-controlled pilot study revealed a mean drop in weight of 4.0 Kg (SD = 4.3), systolic BP by 8.6 mmHg (SD = 18.6), HbA1C by 1.3% (SD = 1.6), and fasting plasma glucose by 4.3 mmol/L (SD = 4.2). However, only four patients completed the intervention [137] and no control group was included.

The mean methodological score was 3, suggesting that for diabetes, the level of evidence is poor. Given the high prevalence and health consequences of type-2 diabetes, there is an urgent need to perform an RCT testing the effects of HRV-B on clinical outcomes in type-2 diabetes.

## 12. Cognitive Impairment and Dementia

The world population is aging with the advancements in medical care. One significant consequence is the rise in the prevalence of dementia and specifically Alzheimer’s disease. Concerning the vagal nerve, the authors of [138] reviewed eight studies and found a negative association between Alzheimer’s disease and HRV. Inflammation plays a pivotal role in the two main pathophysiological processes of Alzheimer’s disease, namely the beta-amyloid plaques and neurofibrillary tangles [139]. Besides the structural and functional changes in the ANS, the number of intracerebral nicotinic receptors remarkably decreases in the elderly [140]. Age-related anatomical and functional cardiac changes, including in the autonomic system, could interfere with the control of cognitive domains [141].

Executive functions (EF) encompass cognitive processes involved in controlling, organizing, and integrating information [142] and also include working memory, cognitive flexibility, and inhibition [143]. EF decline sharply with age and particularly in dementia [144].

Contradictory findings were reported concerning the relationship between HRV and dementia. Studies have found reduced [145], increased [146], and unchanged [147] parasympathetic functions together with increased sympathetic functions in dementia [145]. HRV in AD and mild cognitive impairment (MCI) were evaluated [148], using 24-h ECG monitoring. They found that both LF and HF HRV were significantly lower in AD compared to MCI. In a meta-analysis [138], a greater degree of autonomic dysfunction (reduced HRV) was found in patients with dementia. Scores on cognitive performance (MMSE and MoCA tests) were evaluated in a sample of elderly people [141]; it was found that the cognitive performance was both significantly and positively correlated with the sympathetic system parameters but not with the parasympathetic system parameters. Thus, the relationships between cognitive performance and HRV in the elderly are mixed.

Can HRV-B improve cognitive performance and EF in the elderly? A systematic review was performed to summarize the existing literature on the effects of HRV-B on EF. Nine out of the 16 studies (56%) found improvements in one of the EF measures following HRV-B. Specifically, attention and inhibition were improved by HRV-B [149]. However, no study included in that review was completed with elderly people. In a study on HRV-B completed with elderly people without dementia, HRV-B resulted in decreased anxiety, depression, and improved attention as measured by the Trail Making A test [150]. However, no control group was included.

The methodological score for this category of studies was 3, suggesting that for dementia/cognitive impairment, the level of evidence is poor–medium. Thus, the evidence regarding the effects of HRV-B on EF for certain outcomes (attention and inhibition) is moderate but specifically for elderly people, the level of evidence is poor. To the best of our knowledge, no evidence exists for the effects of HRV-B on elderly people with dementia.

## 13. Summary and Future Directions

The vagal nerve plays a crucial role in predicting a lower risk of many diseases and a better prognosis for such fatal diseases [52,94]. Looking at the vagal index of HRV, observational studies reviewed here show clear protective roles of the vagal nerve in coronary heart disease, diabetes, hypertension, chronic pain, and cancer. Given the prevailing strong evidence linking HRV to the etiology and prognosis of multiple serious diseases, hospitals should consider routinely measuring HRV to estimate patients’ prognoses.

In surgery, while initially post-operative inflammation is protective [151], lack of its regulation could exacerbate tissue damage, lead to shock, multiple organ dysfunction syndromes (MODS), and even to death [152]. A recent study [153] found that in patients undergoing total knee replacement, those with lower pre-surgical SDNN had a worse postoperative inflammatory trajectory than those with high pre-surgical SDNN. Another recent study found that high pre-surgical HF-HRV strongly predicted less postoperative pain in patients with epilepsy [108]. Those findings emphasize the need for using the measurement of HRV as a clinical management tool and exploring the use of HRV-B in improving post-operative recovery as well.

The studies reviewed here also show positive clinical effects of HRV-B on disease outcomes with high methodological evidence in CHD and HT. HRV-BF is an effective psychophysiological intervention with short- and long-term effects in cardiovascular disease rehabilitation programs. Regarding pain, though mostly derived from small-scale samples, several RCTs do provide evidence that HRV-B may reduce pain among children and adults. In contrast, the levels of evidence in cancer are moderate and mostly concern the “symptom cluster”–pain, distress, fatigue, and depression, and subjective outcomes. For cognitive enhancement, the evidence regarding the effects of HRV-B on EF exists for certain outcomes (attention and inhibition) among the elderly population, but to the best of our knowledge, no research evidence exists for elderly demented people. Finally, for diabetes, the level of evidence is poor. Thus, future studies need to test the effects of HRV-B on long-term disease outcomes using RCT designs in pain, diabetes, and dementia.

From an educational perspective, given the epidemiological, interventional, and neuroimmune evidence linking HRV indices as an index of the vagus nerve regarding the diseases reviewed above, teaching neuroimmunology and specifically neuromodulation in medicine, nursing, and biology needs to take place in the coming years. After RCTs substantiate the effects of HRV-B on CHD, cancer, pain, diabetes, and dementia, then we could recommend its use as an evidence-based adjunct treatment for these challenging conditions.

## Supplementary Materials

The following supporting information can be downloaded at: https://www.mdpi.com/article/10.3390/jcm11195927/s1, Table S1: Effects of HRV-B on various clinical outcomes and health conditions.
