# Supplementary material for: Neuromodulation Applied to Diseases: The Case of HRV Biofeedback
Source: J Clin Med. 2022 Oct 8;11(19):5927. doi: 10.3390/jcm11195927 (PMC9571900; doi:10.3390/jcm11195927)
Supplement: Supplementary file 1 [file jcm-11-05927-s001.zip › jcm-1793282-supplementary.pdf]

Table S1. Effects of HRV-B on various clinical outcomes and health conditions.

Disease	Name & Year	Sample	Design	HRVB Treatment	Outcomes	Results	Quality Rating
**Diabetes**	Druschky & Druschky (2014)	17 patients, diabetic neuropathy, 59% women	Pre-post, no control group	5min x 3/day For 8 weeks	HRV, RSA	Reduced HR, & increased LF-HRV	3
	Munster-Segev et al 2017)	7 Type 2 Diabetes, patients. All women.	Pre-post, no control group	Stress reducing biofeedback mobile app, instructed to use 3 times a day.	Weight, BP, HbA1c, fasting plasma glucose (FPG), triglycerides (TG), and 7-point self-monitoring of blood glucose were measured	Decreased weight, BP, HbA1c and fasting glucose	3
**Cancer**	Burch et al (2020)	N = 34 (17 control, 17 intervention) Cancer patients with pain, distress, fatigue, depression, insomnia and PTSD	Randomized, controlled trial	4–6 weekly training sessions until a criterion of HRV coherence was met	Pain, distress, fatigue, depression, insomnia and PTSD	Significant effect was found for improved sleep	4
	Hasuo et al (2020)	N = 50 (25 control 25 intervention) Patients with incurable cancer with sleep disturbances	Open-label, comparative study	One Hospital based HRV-B session followed by daily home-based practice for approximately 2 weeks	Sleep efficiency and LF-HRV	Significant effects were found on improved sleep measures and increasing rate of LF-HR V	5
	Fournié et al (2021)	N = 17 hematological malignancies	Pre-post, no control group	10 supervised 1-hour sessions of HRV-B over 12 weeks and 20min of daily home practice combining 24 supervised sessions of physical exercise	Fatigue, physical functions and adherence	Significant improvement on physical capacity, muscle strength and flexibility, increased rate of LF-HRV	3
	Ozier & Linden (2018)	N = 5 primary brain tumor patients	Pre-post, no control group	8 weekly HRV-B sessions and 20 min of daily home practice	Depression and anxiety	Significant reductions in depression and anxiety	3
	O'Rourke et al (2017)	31 cancer patients	Randomized control trial: HRV-B or a wait-list control	Weekly HRV-B training up to six weeks	HRV coherence, insomnia, pain, fatigue, distress and depression	Increased HRV-coherence and reduced insomnia, fatigue, pain and distress	4
	Gidron et al. (2017)	6 patients with metastatic colon cancer	Matched-controlled pilot study: HRV-B or historical control	3 months of daily HRV-B for 20min	CEA tumor marker levels	CEA declined only in the HRV-B condition	4
**CHD**	Limmer et al (2021)	N = 43 (23 intervention 20 control) MI patients	Randomized controlled trial	12 weeks of HRV-B with home based HRV-B, 3 times/day for 5 min	SDNN and well-being measures	SDNN significantly increased	5
	Nolan et al (2010)	N = 46 (27 intervention 19 active control) All were diagnosed with CHD	Randomized controlled trial	5 X 1.5 HRV-B sessions + brief cognitive behavioral training	HF, Stress, depression	Improved psychological adjustment was significantly associated with HF-index of vagal HR modulation only in the HRV biofeedback group	5

	Yu et al (2018)	N = 134 (75 intervention, 59 control) CAD patients	Randomized controlled trial	6 sessions of HRV-B	Cardiovascular prognosis included all-cause and cardiac readmissions, emergency visits, and mortality. Depression, hostility, breathing rate, HRV and blood pressure	The HRV-B group had fewer all-cause readmissions and all-cause emergency visits the LF-HRV in the HRV-B group increased at post-intervention and at 1-year follow-up	5
	Lin (2015)	N = 127 (60 intervention, 67 control) CHD patients	Randomized controlled trial	6 one-hour sessions of HRV-B	HRV, hostility and blood pressure	Increased SDNN, LF-HRV and decreased hostility	5
	Climov et al (2014)	N = 24 (13 intervention, 11 control) CHD patients	Randomized controlled trial	10 HRV-B sessions, twice a week	SDNN, anxiety, depression, personality trait and blood pressure	Significant increases in the percentage of cardiac coherence, in relation with an increased SDNN index	5
**Pain**	Yetwin et al (2022)	N = 21 (intervention 9, .control 12) Children aged 8–17 with chronic pain	Randomized controlled trial	5 sessions of HRV-B+ home breathing training	Reported pain intensity, health-related quality of life (HRQOL), and anxiety sensitivity	Participants achieved significant reductions in self-reported pain intensity, higher levels of self-reported school functioning	5
	Hallman et al (2011)	N = 24 (12 intervention, 12 control) stress related chronic muscle pain patients	Randomized controlled trial	10 weekly sessions of HRV-B	HRV indices, pain, stress, disability, quality of life, anxiety and depression	Increased resting HRV as well as enhanced reactivity to hand grip and cold pressor tests	5
	Berry et al (2014)	N = 14 (8 Intervention, 6 control) veterans suffering from chronic pain	Randomized controlled trial.	4 weekly sessions of HRV-B	Stress, negative emotions, physical activity, perceived pain, HRV indices	Increased coherence, reduced pain, stress, negative emotions, and physical activity limitation	5
**Hypertension**	Nolan et al (2010)	N = 65 HT patients (35 intervention, 30 control)	Randomized control trial. Control group received autogenic training (AT)	4 weekly + 2 biweeklies 1-hour sessions of HRV-B OR AT.	Blood pressure	Significant effect on ambulatory blood pressure during wakefulness	5
	Lin et al. (2012).	N = 43 individuals with prehypertension	Randomized control trial. 3 Intervention groups: HRV-B group, slow abdominal breathing group, control group	10-session HRV-B protocol or simple slow abdominal breathing protocol conducted over 5 weeks	HRV, blood pressure, respiration, and galvanic skin response (GSR)	Significant increases in SDNN and reductions in SBP and DBP were only found in the HRV-B group	5
	Rau et al (2003)	N = 22 12 participants with high blood pressure and 10 participants with low blood pressure	2 intervention groups: high/low blood pressure. No control	3 individual sessions of biofeedback of the R-wave-to-pulse interval biofeedback (not precisely HRV-B but	Standard arm-cuff blood pressure measurements across the sessions	Participants with high blood pressure achieved significant reductions of DBP from the beginning of the first to the end of the	3

				conceptually similar) within a 2-week period		last session. In contrast, participants with low blood pressure achieved significant increases in DBP	
**Cognitive performance in the elderly**	Jester et al., (2018)	N = 20 elderly patients	Pre-post, no control group	6 sessions of HRV-B, for 3 weeks, twice a week	Anxiety, depression, cognitive functions	Decreases in depression, state and trait anxiety were observed and an increase in attentional skills	3

Note: HRV = heart rate variability; HRV-B = heart rare variability biofeedback; DBP = diastolic blood pressure; SBP = systolic blood pressure; HF = high frequency HRV; LF = low frequency HRV; HR = heart rate.
